# Physical activity levels in locally advanced rectal cancer patients following neoadjuvant chemoradiotherapy and an exercise training programme before surgery: a pilot study

**DOI:** 10.1186/s13741-017-0058-3

**Published:** 2017-02-16

**Authors:** Lisa Loughney, Malcolm A. West, Borislav D. Dimitrov, Graham J. Kemp, Michael PW. Grocott, Sandy Jack

**Affiliations:** 1 Anaesthesia and Critical Care Research Area, NIHR Respiratory Biomedical Research Unit, University Hospital Southampton NHS Foundation Trust, CE93, MP24, Tremona Road, Southampton, SO16 6YD UK; 2 Integrative Physiology and Critical Illness Group, Clinical and Experimental Sciences, Faculty of Medicine, University of Southampton, CE93, MP24, Tremona Road, Southampton, SO16 6YD UK; 3 MedEx Research Cluster, School of Health and Human Performance, Dublin City University, Dublin, Ireland; 4 Academic Unit of Cancer Sciences, Faculty of Medicine, University of Southampton, Southampton, UK; 5 Academic Unit of Primary Care and Population Sciences, Faculty of Medicine, University of Southampton, Southampton, UK; 6 Department of Musculoskeletal Biology and MRC – Arthritis Research UK Centre for Integrated research into Musculoskeletal Ageing (CIMA), Faculty of Health and Life Sciences, University of Liverpool, Liverpool, UK

**Keywords:** Rectal cancer, Neoadjuvant cancer treatment, Physical activity, Exercise, Prehabilitation, Surgery

## Abstract

**Background:**

The aim of this pilot study was to measure changes in physical activity level (PAL) variables, as well as sleep duration and efficiency in people with locally advanced rectal cancer (1) before and after neoadjuvant chemoradiotherapy (CRT) and (2) after participating in a pre-operative 6-week in-hospital exercise training programme, following neoadjuvant CRT prior to major surgery, compared to a usual care control group.

**Methods:**

We prospectively studied 39 consecutive participants (27 males). All participants completed standardised neoadjuvant CRT: 23 undertook a 6-week in-hospital exercise training programme following neoadjuvant CRT. These were compared to 16 contemporaneous non-randomised participants (usual care control group). All participants underwent a continuous 72-h period of PA monitoring by SenseWear biaxial accelerometer at baseline, immediately following neoadjuvant CRT (week 0), and at week 6 (following the exercise training programme).

**Results:**

Of 39 recruited participants, 23 out of 23 (exercise) and 10 out of 16 (usual care control) completed the study. In all participants (*n* = 33), there was a significant reduction from baseline (pre-CRT) to week 0 (post-CRT) in daily step count: median (IQR) 4966 (4435) vs. 3044 (3265); *p* < 0.0001, active energy expenditure (EE) (kcal): 264 (471) vs. 154 (164); *p* = 0.003, and metabolic equivalent (MET) (1.3 (0.6) vs. 1.2 (0.3); *p* = 0.010). There was a significant improvement in sleep efficiency (%) between week 0 and week 6 in the exercise group compared to the usual care control group (80 (13) vs. 78 (15) compared to (69 ((24) vs. 76 (20); *p* = 0.022), as well as in sleep duration and lying down time (*p* < 0.05) while those in active EE (kcal) (152 (154) vs. 434 (658) compared to (244 (198) vs. 392 (701) or in MET (1.3 (0.4) vs. 1.5 (0.5) compared to (1.1 (0.2) vs. 1.5 (0.5) were also of importance but did not reach statistical significance (*p* > 0.05). An apparent improvement in daily step count and overall PAL in the exercise group was not statistically significant.

**Conclusions:**

PAL variables, daily step count, EE and MET significantly reduced following neoadjuvant CRT in all participants. A 6-week pre-operative in-hospital exercise training programme improved sleep efficiency, sleep duration and lying down time when compared to participants receiving usual care.

**Trial registration:**

Clinicaltrials.gov NCT01325909

**Electronic supplementary material:**

The online version of this article (doi:10.1186/s13741-017-0058-3) contains supplementary material, which is available to authorized users.

## Background

Cancer treatment reduces physical fitness, which appears to be worse in those receiving surgery and radiotherapy in combination with chemotherapy than in those receiving radiotherapy or surgery alone (Moros et al. 2010). Changes in fitness are clinically important: neoadjuvant chemo- and chemoradiotherapy (CRT) reduce physical fitness, objectively measured using cardiopulmonary exercise testing (CPET), which is associated with increased in-hospital morbidity following advanced rectal cancer resection (West et al. 2014) and decreased 1-year overall survival following upper gastrointestinal cancer resection (Jack et al. 2014).

Physical fitness is closely connected with physical activity (PA), although relationships of cause and effect are complex. Remaining physically active during and after cancer treatment improves cancer-related fatigue, psychological distress, quality of life, as well as overall survival and reduces the probability of cancer recurrence (Thomas et al. 2014). Increasing PA following cancer diagnosis may reduce the risk of cancer-specific death in people with breast and non-metastatic colorectal cancer (Holmes et al. 2005; Meyerhardt et al. 2006) or death from any cause in non-metastatic colorectal cancer (Meyerhardt et al. 2006). Exercise training during chemotherapy has a significant beneficial effect on tumour progression and chemotherapy efficacy in solid tumours (Jones and Alfano 2013).

For people diagnosed with locally advanced rectal cancer [tumour, node, metastasis (TNM) stage >T3N+ magnetic resonance imaging (MRI) identified circumferential resection margin threatened cancer], the standard treatment is neoadjuvant CRT followed by surgery (Pucciarelli et al. 2009; Wasserberg 2014). The aim of this pilot study was to measure changes in daily PAL in people with locally advanced rectal cancer scheduled to undergo neoadjuvant CRT followed by surgical resection with a curative intent. We aimed to evaluate changes in daily step count (numbers of steps taken) and overall PAL pre- and post- neoadjuvant CRT in all participants in an attempt to quantify the impact of neoadjuvant CRT on PAL. We also aimed to evaluate changes in daily step count and overall PAL at the start and end of a pre-operative 6-week in-hospital exercise training programme, commenced after completion of neoadjuvant CRT, comparing changes with those observed in a usual care control group (no formal exercise intervention). Exploratory aims included observing changes in other PAL variables such as: total energy expenditure (EE) (daily living EE) and active EE (PA-induced EE) PA duration; lying down time; sleep duration and efficiency; and metabolic equivalent (MET) (intensity of PA) in all participants following neoadjuvant CRT and compare changes in the exercise group compared to the usual care control group.

## Methods

### Participants and study design

This prospective pilot, non-randomised, parallel group, interventional controlled trial was a nested study of a clinical trial (West et al. 2015). This pilot study was approved by the North West Liverpool East Research and Ethics Committee (11/H1002/12) and registered with clinicaltrials.gov (NCT01325909). Written informed consent was obtained from all participants. We recruited consecutive participants between March 2011 and February 2014 referred to the Colorectal Multi-Disciplinary Team, aged ≥18 years, with locally advanced (MRI-defined) circumferential resection margin threatened, operable rectal cancer, undergoing standardised neoadjuvant CRT with no distant metastasis and with WHO performance status <2 (Oken et al. 1982) (categorised between 0 (fully active) to 4 (completely disabled, cannot carry out self-care: totally confined to bed or chair). Exclusion criteria were as follows: inability to give informed consent, non-resectable disease, inability to perform CPET or bicycle exercise due to lower limb dysfunction, and participants who declined surgery or neoadjuvant CRT or who received non-standard neoadjuvant CRT. After completing neoadjuvant CRT, participants were allocated to the exercise training group by default. If unable to commit to the exercise schedule (or living >15 miles from the hospital), they were asked to act as contemporaneously recruited controls (no formal exercise intervention) with the same PA monitoring follow-up. Participant characteristics such as age, gender, past medical history, ASA score (the ASA score is a subjective assessment of patients overall health, categorised into five classes: I (healthy fit patient) to V (patient who is not expected to live 24 h without surgery); and WHO status were collected at the baseline visit.

**Fig. 1:**
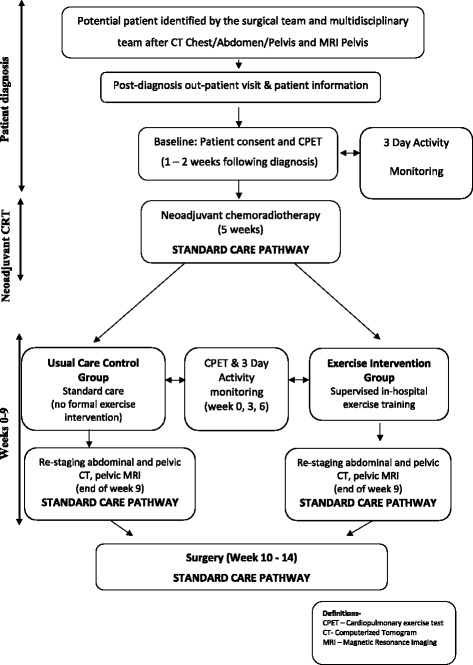
The patient pathway and the time points of assessments

All participants underwent a continuous 72-h period of PA monitoring using SenseWear biaxial accelerometer (Fig. 1). PAL was measured during weekdays at baseline (2 weeks before neoadjuvant CRT), immediately following neoadjuvant CRT (week 0) and at week 3 and week 6. Participants in the exercise training group undertook a 6-week supervised in-hospital exercise training programme (3 sessions per week). The exercise training intensities were responsive to each individual CPET at week 0 and week 3 (informed and altered according to measured work rates at oxygen uptake at lactate threshold and at peak exercise). Exercise training consisted of 40 min (including 5 min warm-up and 5 min cool-down) of interval training on an electromagnetically braked cycle ergometer (Optibike Ergoline GmbH, Germany). The interval training programme consisted of alternating moderate (80% of work rate at oxygen uptake at lactate threshold—4 by 3-min intervals) to severe (50% of the difference in work rates between oxygen uptake at peak and lactate threshold—4 by 2-min intervals) intensities (total 20 min) for the first two sessions. This was then increased to 40 min (6 × 3-min intervals at moderate intensity and 6 × 2-min intervals at severe intensity). The exercise training protocol and procedures are described elsewhere (West et al. 2015).

TNM staging involved flexible sigmoidoscopy for histological diagnosis, colonoscopy, chest, abdomen, and pelvis computer-aided tomography (CT), and 1.5 T pelvic magnetic resonance imaging (MRI). All participants underwent 5 weeks neoadjuvant CRT. Standardised radiotherapy consisted of 45 Gy in 25 fractions on weekdays using a 3D conformal technique with CT guidance. Oral capecitabine (825 mg m^−2^) was given twice daily on radiotherapy days. No participants received brachytherapy. At 9 weeks post-neoadjuvant CRT, participants were restaged using chest, abdomen, and pelvic CT and pelvic MRI. The colorectal Multi-Disciplinary Team was blind to PAL results and participant allocation.

### Measurements

Daily PAL was measured in all participants using a multi-sensor accelerometer (SenseWear Pro® armband; BodyMedia, Inc., Pittsburgh, PADL, USA). The SenseWear Amrband Pro is a reliable estimation of resting EE and provides useful information on daily EE when compared to indirect calorimetry (cancer patients) (Cereda et al. 2007) and reasonable agreement on daily EE when compared with doubly labelled water (free living-adults) (St-Onge et al. 2007). The PA accelerometer was worn on the upper right arm continuously for three consecutive weekdays (except when bathing). Participants in the exercise training group removed the PA monitor during in-hospital exercise training sessions.

The armband estimates EE using measurements from a biaxial accelerometer and sensors that quantify galvanic skin response, heat flux, and skin temperature The device records and reports daily movement: total and active EE, PA duration, number of steps, lying down time, average MET, sleep duration and efficiency (number of minutes of sleep divided by number of minutes in bed). The SenseWear Pro can distinguish between lying down and sleep time by using algorithms that detect the characteristics combination of orientation, motion, temperature and skin conductivity with each state.

### Statistical methods

This was a nested study of a clinical trial, which was powered to detect changes in objectively measured physical fitness (West et al. 2015). Therefore, no a priori formal power calculation was undertaken for such a PA pilot study.

Continuous variables are reported as mean (range), mean (SD) or median and inter-quartile range (IQR), depending on distribution, and categorical variables as frequency (%). The Shapiro-Wilk test for normality of distributions was applied. Descriptive statistics and univariate statistical comparisons of patient characteristics between the groups were undertaken: for continuous variables, a two-sample *t* test when relevant distributional assumptions were met and the Mann–Whitney *U*-test otherwise; for categorical variables, *χ*2 tests or, when cell counts were insufficient, Fisher’s exact test.

Generalised linear mixed models, with a repeated effect for the comparison between the consecutive visits, were used to obtain restricted maximum likelihood (REML) solutions with an unstructured type of the covariance matrix for all or selected measurements in the two groups. Least square means with 95% CIs were obtained. *p* < 0.05 was taken as statistically significant. All analyses were performed with the statistical software IBM SPSS Statistics Ver.22 (IBM Corporation, Armonk, NY, USA).

## Results

A total of 39 participants were recruited of whom 23 (exercise group) and 10 (usual care control group) completed the study (6 participants withdrew consent (dropped out) in the usual care control group: 4 before baseline measurements and 2 during the study). There were significant baseline differences between groups in age, ASA and WHO performance status: the usual care control group were older with poorer subjective performance (Table 1). Further details of participant characteristics are reported elsewhere (West et al. 2015).

**Table 1:** Characteristics of patients scheduled for neoadjuvant cancer treatment and surgery

	Exercise (*n* = 23)	Control (*n* = 10)	*p* value
Age (year)*	64 (45–82)	72 (62–84)	0.015
Gender M:F (%)	15 (65): 8 (35)	8 (80): 2 (20)	0.710
Past medical history^a^	10 (44)	5 (50)	0.617
Heart failure	3 (13)	1 (10)	
Diabetes	2 (9)	1 (10)	
Ischaemic heart disease	5 (22)	3 (30)	
Cerebrovascular disease	0	0	
ASA^b^			0.003
I	11 (48)	0	
II	10 (44)	9 (90)	
III	2 (9)	1 (10)	
WHO performance status^b^			0.035
0	18 (78)	0	
1	5 (22)	9 (90)	
2	0	1 (10)	

Values presented as mean (range). Participants who dropped out of the study are not included in participant characteristics

^a^Frequencies with percentages in parentheses, smoking status assessed as currently smoking: yes (1) vs no (0)

^b^Number of patients (%) WHO performance status and ASA physical status

*p < 0.05 was taken as statistically significant

There was a significant reduction in daily step count between pre-neoadjuvant CRT (baseline) and post-neoadjuvant CRT (week 0) in all participants (4966 (4435) vs. 3044 (3265); *p* < 0.0001), active EE (kcal) (264 (471) vs. 154 (164); *p* < 0.005), and MET (1.3 (0.6) vs. 1.2 (0.3) *p* < 0.05; table 2) (Additional file 1: Table S1 shows overall PAL as mean (SD) and median and inter-quartile range (IQR). Following the 6-week exercise intervention, the exercise group compared to the usual care control group showed significant improvements in sleep efficiency (%) (78 (13) vs. 80 (15) compared to (69 (24) vs. 76 (20); *p* = 0.022), sleep duration (min) (190 (269) vs. 369 (81) compared to (265 (315) vs. 299 (39); *p* = 0.028) and lying down time (min) (360 (352) vs. 47 (476) compared to (541 (360) vs. 341 (372); *p* = 0.029, Table 3) (Additional file 1: Table S2 shows overall PAL data as mean (SD) and median and inter-quartile range (IQR). Note: (1) the exercise training group took the PA monitors off for the duration of each in-hospital exercise session (120 min/week × 6 weeks): (2) sleep efficiency data is presented in only seven participants in the exercise intervention and usual care control group: this is due to an upgrade in software during data collection.

**Table 2:** Pre- and post-neoadjuvant CRT physical activity variables

Physical activity variables	Group	Pre-neoadjuvant CRT	Post-neoadjuvant CRT	Change, % change	*p* value
Step count* (steps/day)	Exercise (*n* = 23)	5705.3 (3746)	3723 (2867)	−2755 (4152), −44 (20)	<0.0001
	Usual care control (*n* = 10)	3701.5 (3569)	2274 (3690)	−4 (2600), −0.1 (78)	
	Overall (*n* = 33)	4966 (4435)	3044.2 (3265)		
MET*	Exercise (*n* = 23)	1.4 (0.5)	1.3 (0.4)	−0.03 (0.3), −2.3 (15)	0.010
	Usual care control (*n* = 10)	1.3 (0.9)	1.1 (0.2)	−0.1 (0.3), −8 (14)	
	Overall (*n* = 33)	1.3 (0.6)	1.2 (0.3)		
Active EE (kcal/day)*	Exercise (*n* = 23)	229 (482.3)	152 (153.7)	−115 (499), −30 (93)	0.003
	Usual care control (*n* = 10)	354 (443.5)	244.3 (198.3)	−223 (861), −47 (70)	
	Overall (*n* = 33)	264.3 (471.3)	154 (163.9)		
PA duration (min/day)	Exercise (*n =* 23)	61 (97.3)	38 (68)	31 (105), 8 (140)	0.45
	Usual care control (*n =* 10)	69 (83)	50 (4)	−34 (151), −41 (52)	
	Overall (*n =* 33)	64 (80.3)	39 (46)		
Lying down (min/day)	Exercise (*n =* 23)	250 (367.3)	360 (351.7)	6 (211), 2 (40)	0.443
	Usual care control (*n =* 10)	351.4 (432.4)	541.3 (360.4)	119 (263), 28 (71)	
	Overall (*n =* 33)	363 (423.9)	483.5 (416.5)		
Sleep efficiency (%)	Exercise (*n =* 7)	78 (9.1)	78 (13)	0.2 (15), 0.3 (21)	0.917
	Usual care control (*n =* 7)	69 (20)	69 (24)	−4 (23), −5 (30)	
	Overall (*n =* 14)	75 (11)	73 (22)		
Sleep duration (min/day)	Exercise (*n =* 23)	220 (330)	190 (269)	0 (141), 0 (35)	0.847
	Usual care control (*n =* 10)	264.5 (284)	265 (315)	143 (235), 56 (85)	
	Overall (*n =* 33)	260 (285)	44 (318)		
Total EE (kcal/day)	Exercise (*n =* 23)	1668 (932)	1701 (921)	−234 (1013), −0.1 (63)	0.33
	Usual care control (*n =* 10)	1867 (833)	1741 (416)	−241 (1019), 7 (147)	
	Overall (*n =* 33)	1668 (846)	1707 (722)		

Values are presented as median (IQR). Absolute change (no brackets) and relative (percentage) change (in brackets) are based on the difference between post-neoadjuvant CRT (week 0) and pre-neoadjuvant CRT (baseline) within each group. All data is averaged over the 72-h period of PA monitoring. Note: due to an upgrade in software during data collection, sleep efficiency is reported in 7/23 (exercise) and 7/10 (usual care control)

*EE* energy expenditure, *PA* physical activity

*p < 0.05 is taken as statistically significant

**Table 3:** Changes in physical activity variables (week 0–week 6)

Physical activity variables	Group	Week 0	Week 3	Week 6	Change, % change	*p* value
Step count (steps/day)	Exercise (*n =* 23)	3723 (2867)	6333 (5291)	5401 (3869)	−1544 (5800), 22 (52)	0.728
	Usual care control (*n =* 10)	2274 (3690)	6422 (7158)	4792 (4370)	1580 (1732), 57 (70)	
MET	Exercise (*n =* 23)	1.3 (0.4)	1.5 (0.4)	1.5 (0.5)	−0.1 (0.6), 7 (38)	0.440
	Usual care control (*n =* 10)	1.1 (0.2)	1.2 (0.3)	1.5 (0.5)	0.2 (2), 17 (174)	
Active EE (kcal/day)	Exercise (*n =* 23)	152 (154)	355 (486)	434 (658)	181 (1228), 46 (92)	0.743
	Usual care control (*n =* 10)	244 (198)	322 (517)	392 (701)	320 (1368), 110 (284)	
PA duration (min/day)	Exercise (*n =* 23)	38 (68)	76 (70)	84 (110)	35 (185), 41 (105)	0.992
	Usual care control (*n =* 10)	39 (46)	66 (89)	89 (132)	85 (243), 100 (276)	
Lying down (min/day)*	Exercise (*n =* 23)	360 (352)	95 (438)	47 (476)	18 (332), 4 (82)	0.029
	Usual care control (*n* = 10)	541 (360)	321 (352)	341 (372)	10 (292), 2 (82)	
Sleep efficiency (%)*	Exercise (*n* = 7)	78 (13)	78 (14)	80 (15)	6 (28), 6 (39)	0.022
	Usual care control (*n* = 7)	69 (24)	66 (14)	76 (20)	6 (11), 7 (17)	
Sleep duration (min/day)*	Exercise (*n* = 23)	190 (265)	405 (70)	369 (81)	0 (141), 1 (52)	0.028
	Usual care control (*n* = 10)	265 (315)	197 (244)	299 (39)	143 (235), 3 (112)	
Total EE (kcal/day)	Exercise (*n* = 23)	1707 (921)	1949 (769)	1869 (924)	−2 (1177), −0.1 (63)	0.701
	Usual care control (*n* = 10)	1741 (416)	1962 (730)	1673 (1169)	147 (2705), 7 (147)	

Values presented as median (IQR). Absolute change (no brackets) and relative (percentage) change (in brackets) at week 6 from baseline (pre-neoadjuvant CRT presented in Table 2) within the groups. All data is averaged over the 72-h period of PA monitoring. Note: due to an upgrade in software at the time of data collection, sleep efficiency is reported in 7/23 (exercise) and 7/10 (usual care control)

*p < 0.05 is taken as statistically significant

## Discussion

This pilot study shows that neoadjuvant CRT significantly reduced daily step count, active EE and MET in people with newly diagnosed locally advanced rectal cancer. Furthermore, neoadjuvant CRT had a generally negative effect on the other exploratory PA variables, although findings were not statistically significant. People who participated in the 6-week in-hospital exercise training programme, in the time interval following neoadjuvant CRT and prior to surgery, showed significant improvements in sleep efficiency, sleep duration and lying down time compared to the usual care control group. Furthermore, the exercise group showed an improvement in daily step count and active EE, although these findings did not reach statistical significance.

It has been previously been reported that neoadjuvant chemo- and chemoradiotherapy significantly reduce physical fitness and this change is associated with post-operative complications and reduced 1-year survival in locally advanced rectal and upper gastrointestinal cancer (West et al. 2014; Jack et al. 2014). However, little is known about its effect on PAL and to our knowledge, we are the first to report daily PAL in people with locally advanced rectal cancer scheduled for neoadjuvant cancer treatment and surgery. PAL is commonly quantified by using MET which is scored as follows: ≥1.70 (active person); 1.40–1.69 (predominantly sedentary); <1.40 (very inactive); and 1.2 (chair- or bed-bound) (Black et al. 1996). We reported a MET score at cancer diagnosis 1.3 (0.6) which significantly reduced to 1.2 (0.3) following neoadjuvant CRT. This MET score suggests that people in our study were predominantly sedentary following neoadjuvant CRT. Although findings were not statistically significant, we reported lying down time at cancer diagnosis 363 (424) min compared to 484 (417) following completing neoadjuvant CRT. We also reported that, at cancer diagnosis prior to commencing cancer treatment, people in our study had a lower than recommended daily step count (7000–10,000) of 4966 steps (4435) which further reduced to 3044 steps (3265) following neoadjuvant CRT. Daily step count reported following CRT in our study is comparable to daily step count reported in people living with Chronic Obstructive Pulmonary Disease (COPD) (Tudor-Locke et al. 2009). Although little is known about low levels of PA in people with cancer, low levels of PA in people with COPD is associated with development of systemic consequences such as skeletal muscle weakness, osteoporosis, cardiovascular disease (Booth et al. 2000) and with hospital admission and mortality (Garcia-Aymerich et al. 2006).

Participation in the exercise programme had a positive influence on PAL outside the programme similar to findings reported in other studies in people with breast cancer who participated in an exercise programme during adjuvant cancer treatment (Campbell et al. 2005; Adamsen et al. 2009). Although findings were not significant, we reported an improvement in active EE and MET following participation in the exercise programme initiated following neoadjuvant CRT and before surgery. We also showed that daily step count 3 weeks following completion of neoadjuvant CRT (week 0) almost doubled in both groups compared to week 0 but further reduced at week 6, more so in the usual care control group (it must be noted, there were no statistical changes in daily step count following participation in the exercise programme therefore caution is required while interpreting our findings). Additionally, following participation in the exercise programme, there was a significant improvement in sleep efficiency (as well as sleep duration and lying down time) which may be clinically important: sleep disturbance in people with cancer is the second most common reported symptom (Cleeland et al. 2013). Sixty-one percent of people with breast cancer undergoing chemotherapy and radiotherapy report having significant sleep problems (measured using Pittsburgh Sleep Quality Index) which is related to poor health-related quality of life (HRQoL) (Fortner et al. 2002). To our knowledge, only one other study in people with breast cancer scheduled for multimodal treatment (surgery and adjuvant cancer treatment) has assessed sleep disturbance in the context of exercise training during cancer treatment (measured using General Sleep Disturbance response scale) (Naraphong et al. 2015). Although findings from this study did not reach statistical significance, there was a decline in sleep disturbance following a 12-week exercise programme.

To date, measures assessing PAL in people with cancer mainly include subjective self-reported measures such as: Short Form Health Survey (SF-36) (Campbell et al. 2005; Mock et al. 2005; Hoffman et al. 2014); Physical Activity Questionnaire (PAQ) (Mock et al. 2005); Scottish Physical Activity Questionnaire (SPAQ) (Campbell et al. 2005); and leisure time physical activity (Adamsen et al. 2009), all of which provide a patient’s personal perception of their daily activities. Such questionnaires have been found to be of limited validity and reliability (Shepard 2003). Patients’ estimations of time spent on activities have been shown to be inconsistent when compared to values recorded using PA monitors (Hoffman et al. 2014). PA monitors have been validated as a measure of PAL in several patient cohorts such as in people with physical disabilities, COPD (Pitta et al. 2005; Rabinovich et al. 2013) and spinal cord injury (Hiremath et al. 2013). PA monitors provide direct measures of specific behaviours such as steps per day (Matthews et al. 2012) as well as the time spent being active (intensity of activity), standing, sitting and lying (Pitta et al. 2005). One recent study reported that cancer patients participating in a lifestyle intervention during chemotherapy reported 366% higher moderate-to-vigorous intensity PA (MPVA) using the International PA Questionnaire compared to measures collected using SenseWear accelerometers (Vassbakk-Brovold et al. 2016). Our study highlights that objective measures of PAL throughout the cancer care journey are worthy of attention: they are relatively simple to undertake and to date have not been used in the perioperative setting.

Strengths of this study include its prospective design, the homogenous study population (only operable locally advanced rectal cancer patients), the clearly defined exercise intervention and the standardised neoadjuvant CRT regime. PA was averaged over a 72-h period, measured in an objective manner using validated SenseWear activity monitors. Furthermore, participants in the exercise group did not wear the PA monitors during exercise sessions. Potential weaknesses of this study include its design as a relatively small pilot study, which was powered to detect changes in objectively measured physical fitness (West et al. 2015), and the limitation of recruitment to one single centre, which may limit generalisability of results. This was a non-randomised design study (i.e. participants in the usual care control group were people who were living >15 miles from the hospital) and there was significant baseline differences between groups in age, ASA and WHO performance status: the usual care control group were older with poorer subjective performance. Furthermore, differences exist in group sample size, 23/23 (exercise) and 10/16 (usual care control) completed the study. Sleep efficiency data were only available for 7 in each group: this was due to an upgrade in software during data collection.

## Conclusions

Our study shows that neoadjuvant CRT significantly reduces MET score, active EE and daily step count in people with locally advanced rectal. People who participated in a 6-week in-hospital exercise training programme following neoadjuvant CRT showed a significant improvement in sleep efficiency, sleep duration and lying down time and an apparent improvement in daily step count and overall PAL compared to the usual care control group.

## Additional file

Additional file 1: Table S1. PA variables pre- and post-neoadjuvant chemoradiotherapy. **Table S2.** PA variables between week 0 (post-noeadjuvant CRT) and week 6. (DOCX 25 kb)

## Abbreviations

COPD: Chronic obstructive pulmonary disease
CPET: Cardiopulmonary exercise test
CRT: Chemo-radiotherapy
CT: Computer-aided tomography
EE: Energy expenditure
HRQoL: Health-related quality of life
IQR: Inter-quartile range
MET: Metabolic equivalent threshold
MRI: Magnetic resonance imaging
PA: Physical activity
PAL: Physical activity levels
PAQ: Physical activity questionnaire
REML: Restricted maximum likelihood
SD: Standard deviation
SF-36: Short form health survey
SPAQ: Scottish physical activity questionnaire
TNM: Tumour, nodes, metastasis
