# Supplementary material for: Physical activity levels in locally advanced rectal cancer patients following neoadjuvant chemoradiotherapy and an exercise training programme before surgery: a pilot study
Source: Perioper Med (Lond). 2017 Feb 16;6:3. doi: 10.1186/s13741-017-0058-3 (PMC5311720; doi:10.1186/s13741-017-0058-3)
Supplement: Additional file 1: Table S1. — PA variables pre- and post-neoadjuvant chemoradiotherapy. Table S2. PA variables between week 0 (post-noeadjuvant CRT) and week 6. (DOCX 25 kb) [file 13741_2017_58_MOESM1_ESM.docx]

APPENDIX 1. **PA Variables Pre- and Post- neoadjuvant chemoradiotherapy**

|  | **Pre neoadjuvant CRT**  median (IQR) | **Pre neoadjuvant CRT**  mean (SD) | **Post neoadjuvant CRT**  median (IQR) | **Post neoadjuvant CRT**  mean (SD) |
| --- | --- | --- | --- | --- |
| **Number of steps (steps/day) ***  Exercise (n=23)  Usual care control (n=10)  Overall (n=33) | 5705.3 (3746)  3701.5 (3569)  4966 (4435) | 5973.1 (3625.2)  5097.1 (4239.1)  5707.6 (3775.7) | 3723 (2867)  2274 (3690)  3044.2 (3265) | 4055.9 (2823.8)  3143 (2111.0)  3799.1 (2642.5) |
| **Total energy expenditure (kcal/day)**  Exercise (n=23)  Usual care control (n=10)  Overall (n=33) | 1668 (932)  1867 (833)  1668 (846) | 1849 (682.7)  2033.1 (709.8)  1904.8 (685.2) | 1700.7 (921)  1741 (416)  1706.4 (722) | 1681.2 (642.0)  1879.3 (385.9)  1736.9 (582.3) |
| **Physical activity duration (min/day)**  Exercise (n=23)  Usual care control (n=10)  Overall (n=33) | 61 (97.3)  69 (83.1)  64 (80.3) | 88.7 (87.3)  99.3 (110.0)  91.9 (93.1) | 38.3 (68.0)  39.2 (45.7)  39.2 (45.7) | 75.7 (95.7)  48.4 (28.2)  68 (82.8) |
| **Lying down (min/day)**  Exercise (n=23)  Usual care control (n=10)  Overall (n=33) | 25 (367.3)  351.4 (432.4)  63 (423.9) | 169.7 (215.1)  293.6 (204.0)  207.2 (216.5) | 36 (351.7)  541.3 (360.4)  83.5 (416.5) | 156.4 (216.4)  398.7 (223.3)  224.5 (241.6) |
| **Active energy expenditure (kcal/day) ***  Exercise (n=23)  Usual care control (n=10)  Overall (n=33) | 229 (482.3)  354 (443.5)  264.3 (471.3) | 445.7 (506.6)  503.5 (580.3)  463.2 (521.5) | 152.0 (153.7)  244.3 (198.3)  154 (163.9) | 341.1 (507.6)  235.6 (137.5)  311.5 (436.0) |
| **Sleep duration (min/day)**  Exercise (n=23)  Usual care control (n=10)  Overall (n=33) | 22 (330)  264.5 (284)  60 (285) | 132.4 (171.3)  193.7 (133.5)  151 (161.3) | 19 (269)  264.7 (315)  43.5 (318) | 117.8 (172.9)  282 (176.9)  164 (186.9) |
| **METS***  Exercise (n=23)  Usual care control (n=10)  Overall (n=33) | 1.4 (0.5)  1.3 (0.9)  1.3 (0.6) | 1.4 (0.3)  1.5 (0.5)  1.5 (0.4) | 1.3 (0.4)  1.1 (0.2)  1.2 (0.3) | 1.3 (0.4)  1.2 (0.2)  1.3 (0.3) |
| **Sleep efficiency (%)**  Exercise (n=7)  Usual care control (n=7)  Overall (n=14) | 78.0 (9.1)  69.2 (20.0)  74.9 (10.6) | 76.5 (6.1)  68.8 (14.8)  72.7 (11.6) | 77.9 (13.3)  68.6 (23.6)  72.8 (22.3) | 78.0 (8.7)  64.4 (13.7)  70.7 (13.2) |

* P<0.05 was taken as statistically significant. All data is averaged over the 72 h period of PA monitoring. Note: due to an upgrade in software at the time of data collection, PAL and sleep efficiency is reported in 7/23 (exercise) and 7/10 (usual care control).

APPENDIX 2. **PA Variables between week 0 (post noeadjuvant CRT) and week 6**

| **Physical Activity Variable** | **Week 0**  Median (IQR) | **Week 0**  Mean (SD) | **Week 3**  Median (IQR) | **Week 3**  Mean (SD) | **Week 6**  Median (IQR) | **Week 6**  Mean (SD) |
| --- | --- | --- | --- | --- | --- | --- |
| **Number of steps (steps/day)**  Exercise (n=23)  Usual care control (n=10)  Overall (n=33) | 3723.0 (2867)  2274.0 (3690)  3044.2 (3265) | 4055.9 (2823.8)  3143.0 (2111.0)  3799.1 (2642.5) | 6333.4 (5291)  6421.9 (7158)  6333.4 (5356) | 6294.5 (2978.4)  6121.3 (3937.9)  6240.3 (3243.2) | 5401.0 (3869)  4792.3 (4370)  5275.5 (3938) | 6477.5 (3374.5)  5796.9 (3592.3)  6264.8 (3400.5) |
| **Total energy expenditure (kcal/day)**  Exercise (n=22)  Usual care control (n=10)  Overall (n=33) | 1668.0 (932)  1867.0 (833)  1668.0 (846) | 1849.0 (682.7)  2033.1 (709.8)  1904.79 (685.2) | 1948.7 (769)  1962.0 (730)  1948.7 (708) | 1990.6 (551.0)  2019.0 (553.7)  1999.5 (543.0) | 1868.5 (924)  1672.8 (1169)  1765.0 (1053) | 2013.2 (846.6)  2188.3 (1054.4)  2067.9 (902.8) |
| **Physical activity duration (min/day)**  Exercise (n=22)  Usual care control (n=10)  Overall (n=33) | 38.3 (68.0)  39.2 (45.7)  39.2 (45.7) | 75.7 (95.7)  48.4 (28.2)  68.0 (82.8) | 75.8 (69.8)  65.7 (88.9)  69.7 (78.9) | 84.4 (64.8)  81.6 (54.2)  83.5 (60.8) | 84.0 (110.3)  89.3 (131.8)  84.0 (110.3) | 94.9 (74.2)  101.0 (68.5)  96.8 (71.4) |
| **Lying down time (min/day) ***  Exercise (n=22)  Usual care control (n=10)  Overall (n=32) | 360 (351.7)  541.3 (360.4)  83.5 (416.5) | 156.4 (216.4)  398.7 (223.3)  224.5 (241.6) | 95.0 (438.1)  320.5 (351.9)  75.0 (416.8) | 174.0 (236.7)  298.3 (198.9)  212.8 (229.9) | 46.5 (476.3)  340.7 (371.6)  74.0 (446.6) | 181.2 (243.1)  299.0 (205.5)  218.0 (235.3) |
| **Active energy expenditure (kcal/day)**  Exercise (n=23)  Usual care control (n=10)  Overall (n=33) | 152.0 (153.7)  244.3 (198.3)  154.0 (163.9) | 341.1 (507.6)  235.6 (137.5)  311.5 (436.0) | 366.0 (486)  322.0 (517.0)  344.5 (473.7) | 413.2 (322.7)  377.0 (275.3)  401.9 (304.6) | 433.7 (658.0)  392.3 (701.1)  433.7 (660.6) | 472.2 (387.5)  484.4 (336.6)  476.0 (367.2) |
| **Sleep duration (min/day) ***  Exercise (n=23)  Usual care control (n=10)  Overall (n=32) | 379 (215)  352 (234)  435 (318) | 387 (126)  351 (124)  164 (186.9) | 405 (70)  201 (208)  630 (317) | 402 (37)  255 (137)  159 (189) | 369 (81)  299 (265)  465 (350) | 374 (47)  350 (141  167 (186) |
| **METS**  Exercise (n=23)  Usual care control (n=10)  Overall (n=32) | 1.3 (0.4)  1.1 (0.2)  1.2 (0.3) | 1.3 (0.4)  1.2 (0.2)  1.3 (0.3) | 1.5 (0.4)  1.2 (0.3)  1.4 (0.5) | 1.6 (0.4)  1.4 (0.4)  1.5 (0.4) | 1.5 (0.5)  1.5 (0.5)  1.5 (0.5) | 1.5 (0.4)  1.7 (1.0)  1.6 (0.6) |
| **Sleep efficiency (%)***  Exercise (n=7)  Usual care control (n=7)  Overall (n=14) | 77.9 (13.3)  68.6 (23.6)  72.8 (22.3) | 78.0 (8.7)  64.4 (13.7)  70.7 (13.2) | 78.1 (14.2)  66.1 (14.4)  70.0 (16.8) | 77.6 (6.8)  64.5 (14.0)  71.1 (12.5) | 80.1 (15.3)  76.1 (20.0)  79.7 (14.7) | 73.9 (16.1)  78.7 (10.7)  76.3 (13.4) |

* P<0.05 was taken as statistically significant. All data is averaged over the 72 h period of PA monitoring. Note: due to an upgrade in software at the time of data collection, PAL and sleep efficiency is reported in 7/23 (exercise) and 7/10 (usual care control).
